# Boosting GSH Using the Co-Drug Approach: I-152, a Conjugate of *N*-acetyl-cysteine and β-mercaptoethylamine

**DOI:** 10.3390/nu11061291

**Published:** 2019-06-07

**Authors:** Rita Crinelli, Carolina Zara, Michaël Smietana, Michele Retini, Mauro Magnani, Alessandra Fraternale

**Affiliations:** 1 Department of Biomolecular Sciences, University of Urbino Carlo Bo, 61029 Urbino, Italy; rita.crinelli@uniurb.it (R.C.); c.zara@campus.uniurb.it (C.Z.); michele.retini@uniurb.it (M.R.); mauro.magnani@uniurb.it (M.M.); 2 Institut des Biomolécules Max Mousseron, Université de Montpellier UMR 5247 CNRS, ENSCM, 34095 Montpellier, France; michael.smietana@umontpellier.fr

**Keywords:** glutathione, pro-glutathione molecules, co-drug, antiviral activity, immunomodulation, *N*-acetyl-cysteine, cysteamine

## Abstract

Glutathione (GSH) has poor pharmacokinetic properties; thus, several derivatives and biosynthetic precursors have been proposed as GSH-boosting drugs. I-152 is a conjugate of *N*-acetyl-cysteine (NAC) and *S*-acetyl-β-mercaptoethylamine (SMEA) designed to release the parent drugs (i.e., NAC and β-mercaptoethylamine or cysteamine, MEA). NAC is a precursor of L-cysteine, while MEA is an aminothiol able to increase GSH content; thus, I-152 represents the very first attempt to combine two pro-GSH molecules. In this review, the in-vitro and in-vivo metabolism, pro-GSH activity and antiviral and immunomodulatory properties of I-152 are discussed. Under physiological GSH conditions, low I-152 doses increase cellular GSH content; by contrast, high doses cause GSH depletion but yield a high content of NAC, MEA and I-152, which can be used to resynthesize GSH. Preliminary in-vivo studies suggest that the molecule reaches mouse organs, including the brain, where its metabolites, NAC and MEA, are detected. In cell cultures, I-152 replenishes experimentally depleted GSH levels. Moreover, administration of I-152 to C57BL/6 mice infected with the retroviral complex LP-BM5 is effective in contrasting virus-induced GSH depletion, exerting at the same time antiviral and immunomodulatory functions. I-152 acts as a pro-GSH agent; however, GSH derivatives and NAC cannot completely replicate its effects. The co-delivery of different thiol species may lead to unpredictable outcomes, which warrant further investigation.

## 1. Introduction

Glutathione (GSH) is the most powerful antioxidant molecule within cells performing a variety of functions that go beyond its protective activity against reactive oxygen species (ROS). Indeed, in its reduced form, GSH not only acts as a scavenger of ROS and as a substrate for antioxidant enzymes, but also promotes drug detoxification [1]. Moreover, by controlling the redox potential, GSH modulates many redox-sensitive proteins within signaling pathways [2]. Notably, GSH itself can be conjugated to the cysteines of proteins in a process known as *S*-glutathionylation. It is widely accepted that thiol redox transitions cause changes in protein activity, abundance, localization, and interaction with other macromolecules [3]. These properties of GSH may at least partially explain its ability to stimulate cell proliferation and act as an immunomodulator, although research in this field is still limited.

Alterations in GSH levels may be transient in response to an oxidative insult, or they may become chronic under conditions of prolonged oxidation and/or dysfunction/deficiency of the enzymes involved in GSH synthesis/degradation. Inborn errors in GSH metabolism include those arising from defective γ-glutamyl-cysteine ligase and glutathione transferase which are the most frequently occurring disorders [4]. Conversely, examples of acquired GSH deficiency include mitochondrial diseases, cystic fibrosis, and many viral/microbial infections [5,6]. In the last few decades, the number of pathologies found to be associated with low GSH levels has risen rapidly, prompting researchers to consider GSH and its derivatives as possible therapeutic agents. Cellular GSH concentration can be affected by the exogenous administration of GSH or GSH-boosting drugs, such as glutathione esters and GSH biosynthetic precursors, which have been used to overcome the poor pharmacokinetic properties of GSH [7,8]. GSH and *N*-acetylcysteine (NAC) have also been used in the co-drug approach as bioconjugates of several therapeutics employed in the treatment of neurodegenerative diseases [9]. In this context, the use of I-152 marks the very first attempt to combine two pro-GSH molecules into one to potentiate both the cellular uptake and to improve the biopharmaceutical properties of the parent drugs.

In this review, the design, metabolism and ability of I-152 to affect GSH levels are discussed. Moreover, the antiviral and immunomodulatory properties of I-152 in light of its GSH-boosting activity are also summarized.

## 2. I-152 Design and Synthesis

**Figure 1:**
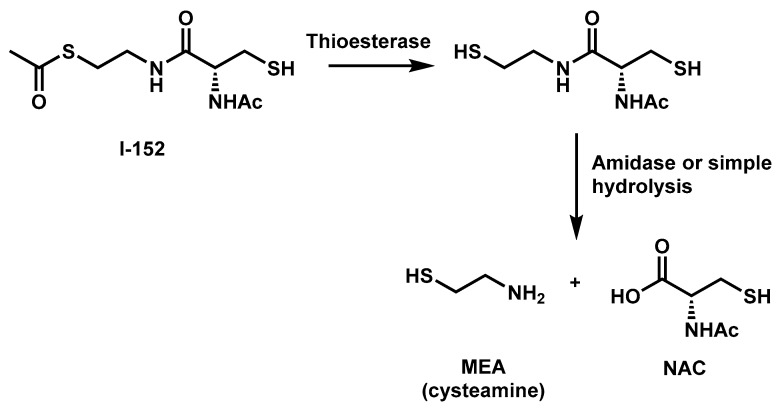
Chemical structure and proposed metabolism of I-152. NAC, *N*-acetyl-cysteine; MEA, β-mercaptoethylamine or cysteamine.

I-152 is a conjugate of NAC and *S*-acetyl-β-mercaptoethylamine (SMEA) linked together by an amide bond. The molecule was synthesized by Oiry et al., in 2001 using commercially available *N*-acetyl-*S*-trityl–L-cysteine and *S*-acetylcysteamine hydrochloride [10]. Experiments performed in cell-free extracts have shown that this compound is deacetylated to the corresponding dithiol derivative, which may be responsible for the in-situ release of NAC and MEA [10] (Figure 1).

In an attempt to enhance the lipophilic properties of the molecule, a series of I-152 analogues carrying different *S*-acyl groups on the MEA moiety and *S*-acylation of the free thiol group were subsequently synthesized by the same team [11]. In terms of pro-GSH activity, the potency of the molecules increased with the presence of the free thiol group. By contrast, the presence of an R-radical on the MEA moiety had no significant effects.

The initial aim of combining NAC and MEA was to design a new potent antioxidant molecule able to liberate two potential pro-GSH compounds after metabolic conversion. NAC is the *N*-acetyl derivative of the natural amino acid L-cysteine; thus, it is a direct precursor of glutathione. NAC has long been used therapeutically as a well-tolerated and safe medication for the treatment of various pathologies, including paracetamol intoxication and cystic fibrosis. Its efficacy as an antioxidant has been demonstrated in a wide range of clinical settings where oxidation is tightly linked to GSH deficiency, given that NAC is a poor direct antioxidant [12]. Although NAC was designed to facilitate membrane permeability, it has been suggested that its pharmacological activity might rely on the reduction of plasma cystine to cysteine which then enters the cells and sustains glutathione synthesis [13]. NAC-induced cytoprotection and the NAC signal transduction pathway are not well understood. Recent evidence suggests that NAC may modulate antioxidant pathways by increasing the expression of miR-141 regulating Keap1/Nrf2 signaling, at least under conditions in which the miRNA is downregulated [14]. β-mercaptoethylamine (MEA) or cysteamine is a product of the constitutive degradation of Coenzyme A. It derives from the cleavage of pantetheine to form MEA and pantothenate (vitamin B5). In mammalian cells, MEA can be oxidized to hypotaurine and taurine by aminothiol dioxygenase [15]. The thiol cysteamine can be oxidized into the disulfide cystamine depending on the local redox environment. Both forms have been used in clinical and experimental settings; however, in most cases their specific role in the observed biological effects was not understood. High intracellular levels of GSH are probably sufficient to reduce cystamine to cysteamine; thus, it has been proposed that most of the effects of cystamine may be mediated by its reduced form [16]. On the other hand, there is evidence that cysteamine has the propensity to form disulfides in vivo, suggesting that cysteamine-containing disulfides such as cystamine may normally be present along with cysteamine in mammalian tissue under physiological conditions [17]. Cysteamine can perform a wide range of functions acting as a cystine-depleting agent, pro-GSH molecule, enzyme inhibitor, and gene expression modulator. Interestingly, many of these activities are thought to rely on thiol/disulfide exchange reactions between cystamine/cysteamine and susceptible protein cysteine sulfhydryl groups in a process called cysteaminylation [17]. Cysteamine, in the form of cysteamine bitartrate (Cystagon), has been approved by the Food and Drug Administration (FDA) for the clinical treatment of nephropathic cystinosis acting as a cystine-depleting agent by forming cysteine-cysteamine mixed disulfides [18]. Recent studies suggest that cysteamine may have several other potential therapeutic applications beyond cystinosis, including the treatment of neurodegenerative diseases and cancer. Indeed, cysteamine/cystamine is an efficient inhibitor of transglutaminase and caspase 3 which play a pivotal role in mutant Huntington protein processing [19]. Other proteins whose activity has been reported to be affected by cysteamine/cystamine treatment are protein kinase C and metalloproteinases, important drug targets in cancer progression and metastasis [20,21]. More poorly understood are the mechanisms mediating the pro-GSH activity of cysteamine and its oxidized form. Cysteamine treatment of normal and cystinotic cells has been shown to increase GSH content. However, the authors claimed that the increase of cysteine levels resulting from cystine reduction can only partially explain elevated GSH levels [22]. The ability of cystamine and, less potently, of cysteamine to activate the Nrf2 antioxidant pathway was shown by Calkins et al., in astrocytes [23]. Cystamine-mediated activation of Nrf2 was found to be inversely correlated with the GSH content of the culture and the increase in GSH levels was shown to be dependent on de novo synthesis. Unfortunately, experiments performed with Nrf2 knockout cells revealed that the cystamine-induced GSH increase was Nrf2-independent. Hence, cystamine-induced GSH up-regulation seems to involve other yet-unknown mechanism(s).

## 3. I-152: Metabolism and Effects on GSH Levels

The ability of I-152 to increase basal intracellular GSH content was evaluated in human and murine cellular models. In primary human monocyte-derived macrophages (MDMs) and other human cell lines, I-152 ranging from 10 µM to 1 mM was found to increase GSH content even at doses which were ineffective when NAC and MEA, alone or in equimolar combinations, were used [10]. In murine monocyte/macrophage-like cell line RAW 264.7 and macrophages obtained from the peritoneal cavity of mice, low concentrations of I-152, i.e., 1 mM, increased GSH level [24]. By contrast, high I-152 concentrations (i.e., 10 and 20 mM) caused GSH depletion but provided large quantities of I-152 and NAC which can be used to resynthesize GSH [24]. The decrease in the GSH content at high I-152 doses has been suggested to be the consequence of a negative feedback of GSH on γ-glutamylcysteinyl synthetase, the first enzyme involved in GSH synthesis [10]. Alternatively, GSH could be depleted because of its conjugation to I-152 in a reaction catalyzed by the detoxification enzyme glutathione-*S*-tranferase (GST). Preliminary in-vitro experiments indicate that GSH could be indeed conjugated to I-152 by GST. Under the condition of GSH depletion, cysteamine could be converted into cystamine, which has been shown to inhibit γ-glutamylcysteinyl synthetase, further supporting the decrease in GSH levels [25].

Less information is available on I-152 in-vivo metabolism. Preliminary studies were performed in our laboratory to assess the distribution of I-152 and its metabolites in mouse organs after I-152 intraperitoneal injection (i.p.). GSH and cysteine levels were also measured. The analyses were performed in selected organs, i.e., the lymph nodes, brain and pancreas. The lymph nodes were chosen because it has been reported that lymphoid tissues are significantly more ‘‘reduced’’ than the other tissues, and that the immune response is strongly influenced by variations in the redox state [26]. In particular, some lymphocyte functions, such as DNA synthesis, are favored by high levels of GSH, while other redox-sensitive pathways are favored by low intracellular GSH. However, immunological functions in the diseases characterized by oxidative stress can be restored by cysteine or GSH supplementation [27,28]. The brain generates high levels of ROS due to its high oxygen consumption, hence it is more susceptible to the damaging effects of ROS than other tissues [29,30]. The GSH concentration has a key role in maintaining redox balance in the central nervous system (CNS) and it is altered in neurodegenerative diseases [5,31,32] such as Alzheimer’s disease (AD), Parkinson’s disease (PD), amyotrophic lateral sclerosis (ALS), multiple sclerosis (MS), HIV-associated neurocognitive disorder (HAND or “NeuroAIDS”), cerebral ischemia/reperfusion injury (I/R), and traumatic brain injury (TBI) [33]. The presence of the tightly regulated blood brain barrier (BBB) represents a serious obstacle to effective treatment of CNS disorders, in fact its selective permeability prevents most bioactive molecules from entering the brain [34]. Several antioxidants, including vitamin E (the important scavenger of lipid peroxidation in the brain), vitamin C (intracellular reducing molecule), coenzyme Q10 (transporter of electrons in the electron transport chain, ETC), and NAC (acting as a precursor of GSH) have been used as therapeutic agents. Although antioxidant therapies have shown benefits in preclinical animal models, negative results have been obtained from clinical trials [30]. Moreover, in such treatments, dosage and additives as well as synergistic interactions with other antioxidants must be considered [35]. Hence, the design and development of antioxidant-based therapies for the brain require a great deal of effort [33,34,36]. Lastly, the pancreas plays a major role, along with several other organs, in GSH metabolism, as shown by the high concentration of the tripeptide, its rapid turnover rate, and the presence of high levels of various enzymes involved in GSH metabolism [37]. Therefore, the pancreas requires a great amount of cystine/cysteine for pancreatic enzymes, which is provided by glutathione (GSH). Moreover, the induction of CYP450 enzymes by xenobiotics in pancreatic acinar cells can cause a decrease in GSH content, which can affect both detoxification and pancreatic enzyme synthesis [38].

Our preliminary data indicate that I-152 can be used to deliver precursors for GSH synthesis, in the form of NAC and MEA, to different organs, including the brain (Table 1).

**Figure 2:**
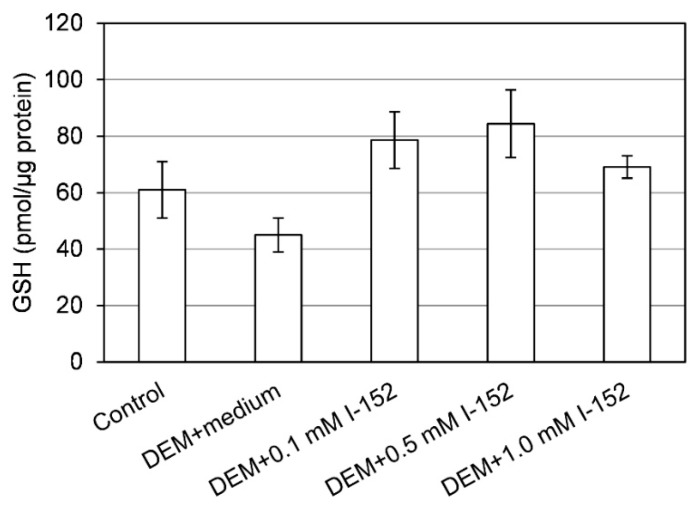
In-vitro replenishment of intracellular GSH by I-152. RAW 264.7 cells were treated with 6 mM diethyl maleate (DEM) for 15 min, then medium not containing I-152 (DEM+medium) or containing I-152 at different concentrations were added for 2h. Reduced glutathione (GSH) content was determined by HPLC [24]. Results represent the mean ± S.D. of two independent experiments.

The presence of NAC within tissues supports the hypothesis that I-152 is able to reach the target site and release in situ the active molecules. In fact, it has been demonstrated that NAC does not pass the cell membrane, but rather reacts with cystine reducing it to cysteine, which then enters the cells and sustains GSH synthesis [13]. Hence, increased cysteine found in the organs can derive from NAC released by I-152 or by the reduction of plasma cystine to cysteine. The presence of NAC in the brain is an interesting aspect since NAC’s ability to cross the BBB is disputed [40]. Hence, I-152 could be a promising pro-drug to release NAC in the brain. Interestingly, increased intracellular thiol availability did not enhance intracellular GSH with the exception of the brain, probably because these cells had normal GSH levels. Indeed, under conditions of GSH depletion, e.g., in cells treated with diethyl maleate (DEM) [41], I-152 even at low concentrations (0.1 and 0.5 mM) exerts a GSH-replenishing effect (Figure 2).

**Figure 3:**
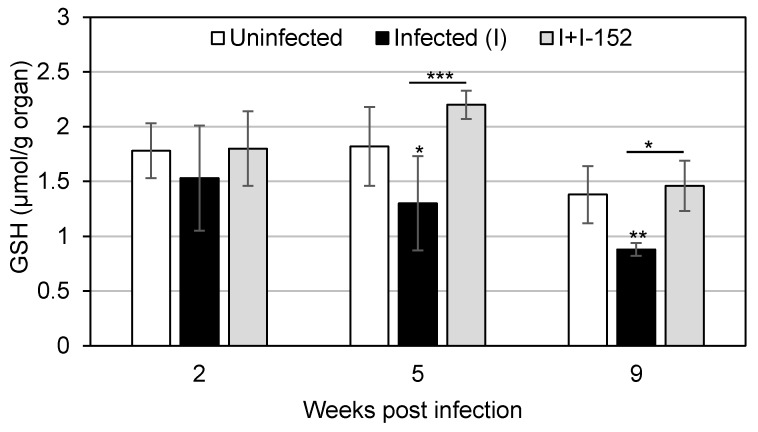
Replenishment of reduced glutathione (GSH) by I-152 in LP-BM5-infected mice. C57BL/6 mice were infected and treated with I-152 (30 μmol/mouse) three times a week, every other day, for a total of 9 weeks. At 2, 5 and 9 weeks after virus inoculation, GSH content in the lymph nodes was determined by HPLC [39,42]. Data are the mean ± S.D. of at least three mice. *p < 0.05; **p < 0.01; ***p < 0.001 (ANOVA). [42]; (unpublished results).

The capacity of I-152 to restore the intracellular GSH content was also studied in vivo in organs of mice experimentally infected with the retroviral complex LP-BM5. This infection causes a disease with many similarities to human Acquired Immuno Deficiency Syndrome (AIDS), including GSH deficiency [42,43]. Glutathione depletion was found in most organs of LP-BM5-infected mice both in the early phases of the disease and later, in particular in the lymphoid organs, e.g., lymph nodes, known to be the site where viral loads are higher [42]. The effect of intraperitoneal administration of I-152 in infected mice is shown in Figure 3, where it can be observed that the treatment was able to re-establish the content of the tripeptide in the lymph nodes of infected mice at all the times of infection. Notably, GSSG content was unaffected by I-152 treatment [42]. Hence, as hypothesized above, I-152 could be used to increase intracellular GSH during aging and various disease states in which antioxidant defense systems can be altered leading to progressive oxidative damage and subsequent cell death and/or significant impairment of several cellular processes.

## 4. Antiviral and Immunomodulatory Properties of I-152

GSH depletion characterizes several viral infections and associated-disease progression [44]. Numerous studies have demonstrated that the use of GSH is effective in reducing viral production in different experimental models suggesting that the administration of the tripeptide can be considered a useful strategy to hinder viral infection and infection-associated symptoms [2,28,44,45]. Due to the poor pharmacokinetic properties of GSH, high GSH concentrations are necessary to sufficiently increase its content in order to obtain an antiviral effect [7,8]. Hence, taking advantage of the GSH-replenishing capacity of I-152, antiviral effects of the molecule were explored in two retroviral infections associated with systemic and tissue decrease in the GSH content, i.e., HIV and LP-BM5 infections. In HIV-1/BaL-infected MDMs, 150 µM I-152 was able to inhibit viral replication by 90% likely interfering with both early and late steps of the virus life cycle [11]. In LP-BM5-infected mice I-152, when administered at a concentration of about 10 times lower than GSH, significantly reduced murine AIDS (MAIDS) symptoms were observed, i.e., splenomegaly and lymphadenopathy, as well as BM5d proviral DNA in spleen and lymph nodes [46]. Actually, the exact mechanisms through which I-152 can exert antiviral activity are not known. However, since I-152 treatment replenished virus-induced GSH depletion both in human MDMs [10,11] and in mouse lymphoid organs (Figure 3), it could be hypothesized that the antiviral effect observed is dependent on GSH. Indeed, GSH can inhibit the replication of viruses by different modes of action [44,45]. For example, it has been reported that administration of GSH permeable analogue GSH-C4 can interfere with the maturation of influenza virus glycoproteins modifying the activity of the host-cell protein disulphide isomerase (PDI) which is essential for the correct disulphide bond formation of viral proteins [47]. Glutathione can interfere with the entry of rhinovirus by inhibiting rhinovirus induction of intercellular adhesion molecule-1 (ICAM-1) mRNA in respiratory epithelial cells [48]. Furthermore, GSH by counteracting the action of reactive oxygen intermediates (ROI), can prevent the activation of NF-kB and HIV replication [49].

I-152 effects against HIV and LP-BM5 can be due to its direct antiviral action, but also to its immunomodulatory activity. In fact, many studies have correlated altered GSH levels with an impaired immune response, suggesting a combination of the highly active antiviral therapy with the GSH replenishment approach [50,51]. Recently, the main functions of GSH in the immune response have been reviewed [52]. Accordingly, a possible immunomodulatory role of I-152 was investigated. In particular, the role of I-152 in Th1/Th2 polarization was studied. In fact, several studies have underlined the correlation between altered GSH levels and an unbalanced Th1/Th2 immune response in favor of Th2 linked to an impaired cytokine production by antigen-presenting cells [42,50,53,54,55,56]. The immunomodulatory activity of I-152 was demonstrated in in-vitro systems where the molecule-stimulated IL-27 p28 gene expression and sustained STAT-1-mediated IRF-1 de novo synthesis [24]; moreover, in vivo, it enhanced Th1 response in ovalbumin immunized mice as well as drove Th1 immune responses and CTL activity against HIV antigens [55,56]. Finally, I-152 treatment, by inducing Th1 cytokine production, restored a balanced Th1/Th2 response in mice affected by murine AIDS [42]. Hence, the effect exerted by I-152 on MAIDS can be derived from its dual mechanisms of action: On the one hand, it can directly inhibit viral replication; on the other hand, it can re-establish a correct GSH content favoring the production of cytokines which induce the Th1 immune response (Table 2).

However, the immunomodulatory activity of I-152 could go beyond the regulation of Th1/Th2 response. In fact, I-152 treatment inhibited total IgG secretion in LP-BM5 infected mice [46] and influenced IgG1/IgG2a ratio in favor of the IgG2a subtype (unpublished results). This second effect is likely the consequence of Th1/Th2 response regulation; in fact, the induction of high IgG1 titers is considered indicative of a Th2-type immune response while high IgG2a are typical of a Th1-type response [57,58]. On the contrary, the inhibition of hypergammaglobulinemia could be the consequence of a lower viral load, but it cannot be excluded that I-152 could also directly affect plasma cell maturation and Ig folding/secretion.

One of the features characterizing murine retrovirus LP-BM5-induced MAIDS is decreased T- and B-cell responses. I-152 treatment was demonstrated to partially restore T and B cell proliferative capacity to mitogens [46]. In this context, Green et al., who had previously demonstrated that monocytic myeloid-derived suppressor cells (M-MDSCs) suppress both T and B cell responses [59,60], found that I-152 could interfere with the suppressive function of M-MDSCs (Green, personal communication). On the whole, these observations suggest that I-152 can influence different components of the immune system. Future studies aimed to shed light on the molecular mechanisms modulated by I-152 will provide further insights into the immunomodulatory activity of this pro-GSH molecule.

## 5. Conclusions

The data presented in this review show that I-152 is an excellent co-drug able to generate NAC and MEA which can be used to increase intracellular GSH. I-152 has been shown to regulate the intracellular GSH content differently depending on the concentration used and the pre-existing redox states; consequently, different redox-sensitive pathways could be activated or inhibited. For example, it has been reported that different concentrations of NAC cause opposite effects on the GSH/GSSG ratio and on the production of pro-inflammatory cytokines [61,62]. Moreover, pro-GSH molecules such as NAC and MEA could affect cellular processes independently of their ability to influence intracellular GSH/GSSG balance [63,64]. Furthermore, the exact mechanisms through which NAC and MEA can increase GSH levels are not yet completely understood. Preliminary experiments have shown that I-152 can influence different redox-signaling pathways linked to GSH [24] and that NAC and MEA administered alone or in combination are less efficient in raising intracellular GSH [10]. Hence, it would be interesting to investigate all of these aspects. Moreover, such studies could yield useful information for the design and synthesis of new redox-modulating agents with improved activity.

## Figures and Tables

**Table 1:** Thiol content in different mouse organs after i.p. administration of I-152.

Time from Injection (min)	30				240			
Thiol Species	NAC	MEA	GSH	Cysteine	NAC	MEA	GSH	Cysteine
BRAIN	+	+	=	=	/	/	↑	↑
LYMPH NODES	+	+	↓	↑	+	/	=	=
PANCREAS	+	+	=	=	/	/	=	=

CD-1 mice were i.p. injected with I-152 (30 µmol/mouse), and the thiol content was determined by HPLC [39]. (+) detectable; (/) undetectable; (↑) increased, (↓) decreased and (=) unchanged compared to control levels. NAC, *N*-acetyl-cysteine; MEA, cysteamine; GSH, reduced glutathione.

**Table 2:** Antiviral and immunomodulatory effects of I-152 in MAIDS.

DISEASE PARAMETERS	INFECTED	INFECTED+I-152	
Splenomegaly	+++	+	[42]
Lymphadenopathy	+++	+	[42]
Hypergammaglobulinemia	+++	++	[42]
Proviral DNA in lymphoid organs	+++	+	[42]
B and T cell proliferative index decrease	+++	++	[42]
Th1/Th2 unbalance in favour of Th2	+++	+	[46]

C57BL/6 mice were infected with the LP-MB5 murine leukemia virus stock and treated with I-152. +, minor; ++, moderate; +++, severe.
